# Patient-Centered Network of Learning Health Systems: Developing a resource for clinical translational research

**DOI:** 10.1017/cts.2016.11

**Published:** 2017-02-02

**Authors:** L. J. Finney Rutten, A. Alexander, P. J. Embi, G. Flores, C. Friedman, I. V. Haller, P. Haug, D. Jensen, S. Khosla, G. Runger, N. D. Shah, T. Winden, V. L. Roger

**Affiliations:** 1 Department of Health Sciences Research, Mayo Clinic, Rochester, MN, USA; 2 Robert D. and Patricia E. Kern Center for the Science of Health Care Delivery, Mayo Clinic, Rochester, MN, USA; 3 Department of Biomedical Informatics, Ohio State University, Columbus, OH, USA; 4 Department of Health Policy Research, Medica Research Institute, Minnetonka, MN, USA; 5 Department of Learning Health Sciences, University of Michigan, Ann Arbor, MI, USA; 6 Essentia Institute of Rural Health, Essentia Health, Duluth, MN, USA; 7 Homer Warner Center for Informatics Research, Intermountain Healthcare, Salt Lake City, UT, USA; 8 Olmsted County Public Health Services, Rochester, MN, USA; 9 Department of Medicine, Mayo Clinic, Rochester, MN, USA; 10 Center for Clinical and Translational Science, Mayo Clinic, Rochester, MN, USA; 11 Department of Biomedical Informatics, Arizona State University, Tempe, AZ, USA; 12 Clinical Research Informatics and Analytics, Allina Health System, Minneapolis, MN, USA; 13 Division of Cardiovascular Diseases in the Department of Internal Medicine, Mayo Clinic, Rochester, MN, USA

**Keywords:** Learning health system, translational research, Patient-Centered Clinical Research Network

## Abstract

**Introduction:**

The Learning Health System Network clinical data research network includes academic medical centers, health-care systems, public health departments, and health plans, and is designed to facilitate outcomes research, pragmatic trials, comparative effectiveness research, and evaluation of population health interventions.

**Methods:**

The Learning Health System Network is 1 of 13 clinical data research networks assembled to create, in partnership with 20 patient-powered research networks, a National Patient-Centered Clinical Research Network.

**Results and Conclusions:**

Herein, we describe the Learning Health System Network as an emerging resource for translational research, providing details on the governance and organizational structure of the network, the key milestones of the current funding period, and challenges and opportunities for collaborative science leveraging the network.

## Introduction

The Patient-Centered Network of Learning Health Systems (LHSNet) Clinical Data Research Network (CDRN) is 1 of 13 CDRNs assembled to create, in partnership with 20 Patient-Powered Research Networks (PPRN) [1], a National Patient-Centered Clinical Research Network (PCORnet) to support pragmatic trials and comparative effectiveness research. The PCORnet was assembled under the auspices of the Patient-Centered Outcomes Research Institute (PCORI) as a distributed research network to support observational and interventional comparative effectiveness research across participating CDRNs, PPRNs, and external contributors [2, 3–5].

The LHSNet comprises 9 participating organizations and includes data on nearly 10 million patients. The LHSNet (http://www.lhsnet.org) Web site provides a map illustrating the geographic locations of the participating sites and describes each of the partnering organizations in the network [6]. The LHSNet includes 6 health systems (Mayo Clinic, Allina Health System, Essentia Health, Intermountain Health Care, University of Michigan, and Ohio State University); 1 health plan (Medica Research Institute); 1 data partner based in a university (Arizona State University); and 1 local public health department (Olmsted County Public Health Services).

A key distinctive feature of LHSNet is its commitment to integrate, in 1 network, health systems, public health offices, and payers to operationalize the vision of a learning health system (LHS) [7]. As defined by the National Academy of Medicine, an LHS is one “in which progress in science, informatics, and care culture align to generate new knowledge as an ongoing, natural by-product of the care experience, and seamlessly refine and deliver best practices for continuous improvement in health and healthcare [8].” The partners in LHSNet represent a diversity of health-care stakeholders, and their collaboration within 1 network uniquely positions the LHSNet to experience and address the challenges of deploying the LHS vision [7].

**Fig. 1:**
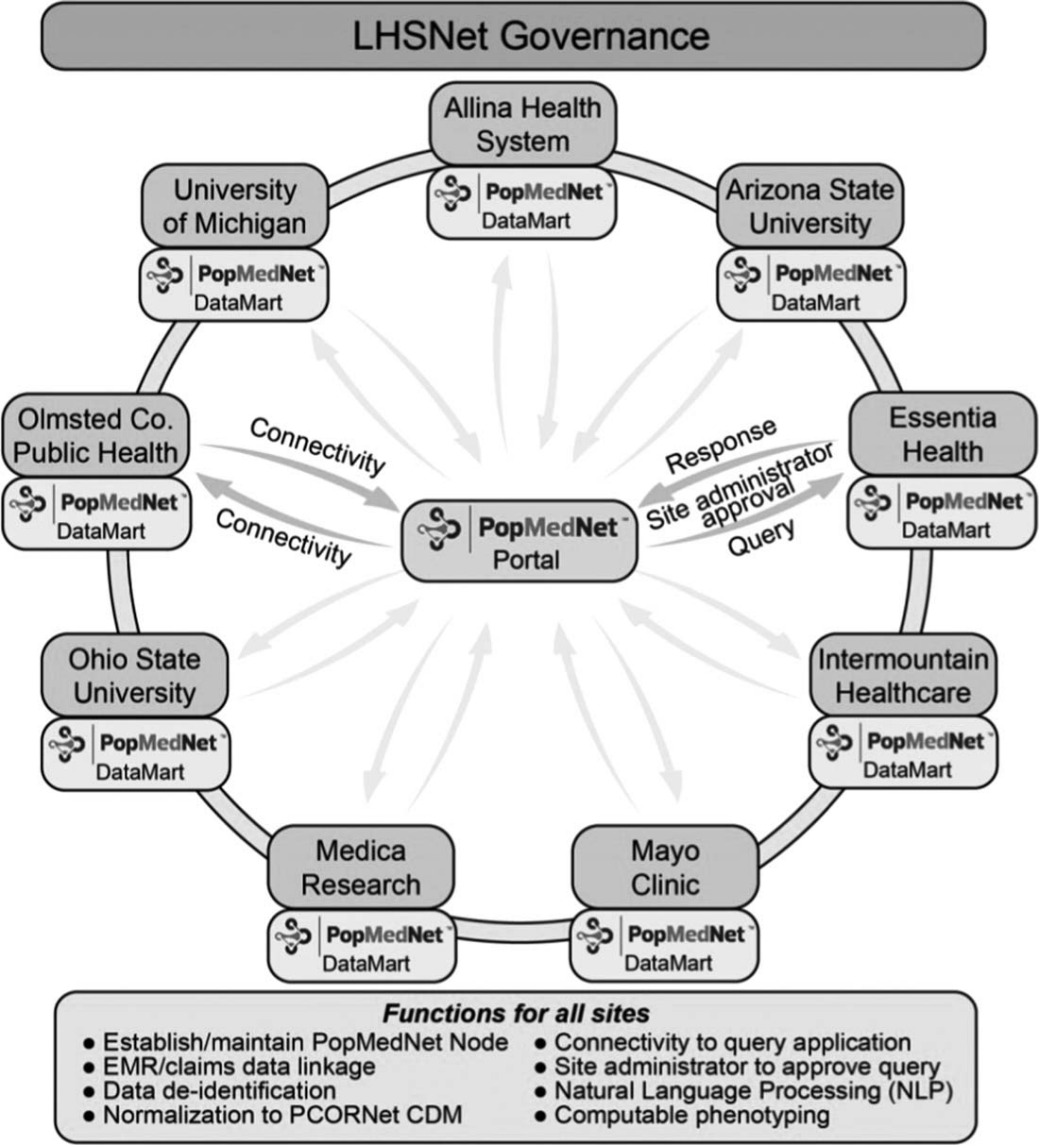
Patient-Centered Network of Learning Health Systems (LHSNet) technical structure. CDM, Common Data Model; Co., County; EMR, Electronic Medical Record; PCORnet, Patient-Centered Clinical Research Network.

The LHSNet leverages existing infrastructure, health information technologies, and data standards to connect its 9 sites and to build the foundation to facilitate patient-centered outcomes research; support large pragmatic clinical trials, and observational and interventional comparative effectiveness studies embedded within the health-care systems; and enable the dissemination, implementation, and evaluation of clinical and community efforts to improve population health. LHSNet is configured as a distributed data model (Fig. 1) wherein each participating site hosts a local Common Data Model (CDM) including standardized data elements common across the LHSNet and PCORnet. Specifically, the distributed data model requires each site to transform their data locally to conform to the CDM, which enables execution of standardized computer programs or data queries to run identically at each of the participating sites. This approach does not require sharing of data in a central repository and provides each participating site in LHSNet complete autonomy over access to and use of their data. Each site has connectivity to the PopMedNet Portal enabling sites to respond to data queries from the PCORnet Distributed Research Network Operations Center. This brief commentary describes the LHSNet partners, LHSNet governance and organizational structures, key milestones of the current funding period, and challenges and opportunities on the horizon.

## LHSNet Partners

The 9 LHSNet partners represent an array of health-care delivery system stakeholders. Table 1 summarizes the partnering organizations and the initial counts of the population of patients with electronic health record (EHR), administrative claims, or registry data available from September 1, 2013 through August 30, 2015 within each of the organizations. Within this preliminary timeframe, the partnering organizations have EHRs for a total of 9,418,988 patients. Currently, the LHSNet sites are developing research data sets that will reflect clinical care for patients seen from January 1, 2013 through December 31, 2015. The demographic characteristics of our network population are similar to that of the overall US population (Table 2).

**Table 1:** Patient-Centered Network of Learning Health System partners

Sites	Population	Headquarters (geographic coverage)
LHSNet, http://www.lhsnet.org/	9,418,988	Rochester, MN (AZ, GA, IA, ID, FL, MI, MN, ND, NE, OH, SD, UT, WI)
Allina Health System, http://www.allinahealth.org/	1,318,178	Minneapolis, MN (MN, WI)
AZ State University, http://www.asu.edu/	3,100,000	Phoenix, AZ (AZ)
Essentia Health, http://www.essentiahealth.org/	514,408	Duluth, MN (MN, WI, ND, ID)
Intermountain Health Care, https://intermountainhealthcare.org/	1,814,329	Salt Lake City, UT (UT, ID)
Mayo Clinic, http://www.mayoclinic.org/	1,395,126	Rochester, MN (MN, IA, WI, GA, FL, AZ)
Medica Research Institute, http://www.medicaresearchinstitute.org/	NA	Minnetonka, MN (MN, WI, ND, SD, IA, NE)
OH State University, https://www.osu.edu/	513,665	Columbus, OH (Central, OH)
Olmsted County Public Health Services, https://www.co.olmsted.mn.us/ocphs	12,108	Rochester, MN (Olmsted County, MN)
University of MI, https://www.umich.edu/	751,174	Ann Arbor, MI (MI)

LHSNet, Learning Health Systems Network; MN, Minnesota; AZ, Arizona; GA, Georgia; IA, Iowa; ID, Idaho; FL, Florida; MI, Michigan; ND, North Dakota; NE, Nebraska; OH, Ohio; SD, South Dakota; UT, Utah; WI, Wisconsin.

**Table 2:** Demographic characteristics of Learning Health Systems Network (LHSNet) population and US census population*

Demographic characteristics	LHSNet	US census^†^
Age (%)		
Under age 18 ythroughout	25.5	23.1
18–64 y	60.2	62.4
65 y and older	14.3	14.5
Sex (%)		
Female	50.2	50.8
Male	49.8	49.2
Race (%)		
American Indian/Alaska Native	2.0	0.9
Asian	2.2	5.4
Native Hawaiian/Pacific Islander	0.5	0.2
Black or African American	6.2	13.2
White	82.9	77.4
Other or unknown	6.1	3.3
Ethnicity (%)		
Hispanic	13.6	17.4
Non-Hispanic	86.4	82.6

* Number of patients/participants with data in electronic health records, claims, or registry from September 1, 2013 through August 30, 2015.

† US Census Bureau, 2014 population estimates.

Members of LHSNet have been collaborating for more than a decade and have strong relationships with patients and communities, academic medical centers, universities, health-care systems, and payers across the United States. Three Clinical and Translational Science Award recipients participate in the LHSNet (Mayo Clinic, University of Michigan, and Ohio State University); all 3 institutions have a long-standing history of strong research collaboration and community engagement. The Clinical and Translational Science Awards at Mayo Clinic, Ohio State University, and the University of Michigan established processes to expedite Institutional Review Board (IRB) approval for multisite trials by creating IRB reciprocity agreements as part of the Midwest Area Research Consortium for Health, a consortium of upper-Midwest Clinical and Translational Science Awards. This experience formed the basis for the LHSNet-wide expansion of the IRB reliance model.

Five of the LHSNet sites such as Allina Health System, Essentia Health, Medica Research Institute, Mayo Clinic, and Olmsted County Public Health Services are also members of the Midwest Research Network (http://midwestresearchnetwork.org/). The Midwest Research Network was founded in 2011 by a core group of research and clinical organizations in the Midwest to support collaborative grant proposals and resource sharing for population health research. The history of collaboration among these groups provides a foundation of trust and cross-institutional knowledge upon which the LHSNet is built.

Essentia Health and Medica Research Institute are also members of the Health Care Systems Research Network (http://www.hcsrn.org/en/). This national collaborative of research departments and health-care systems includes nearly 2000 scientists and research staff with multidisciplinary content and methodological expertise. The extensive experience in multisite research efforts and data sharing of the Health Care Systems Research Network serves as a rich resource for the LHSNet.

The LHSNet is also benefited by the experience and expertise of members of the High Value Health Care Collaborative (HVHC). Intermountain Healthcare and Mayo Clinic are both founding members of the HVHC (https://www.highvaluehealthcare.org/), which is a learning network of delivery systems dedicated to data-driven improvement to deliver high-value care. The primary aims of the HVHC, to evaluate, disseminate, and facilitate the adoption of high-value care models in health-care settings, are closely aligned to the goals of LHSNet. Thus, the experience gained by our members in HVHC greatly informs the planning and execution of LHSNet efforts.

The LHSNet sites have extensive experience working within networks and consortia to study and improve health-care delivery. In addition, LHSNet is developing active partnerships with other CDRNs (Chicago Area Patient-Centered Outcomes Research Network, New York City CDRN) and with PPRNs (Health eHeart Alliance, Mood Patient-Powered Research Network, Alzheimer’s PPRN, and NephCure Kidney Network) to collaboratively conduct research.

## Governance and Organization

**Fig. 2:**
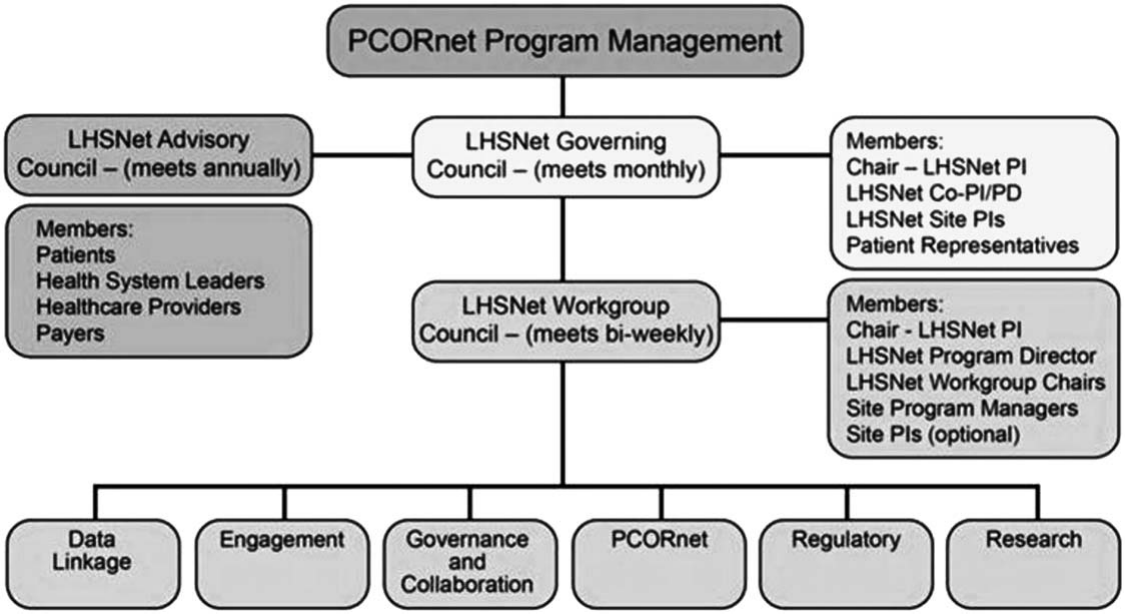
Patient-Centered Network of Learning Health Systems (LHSNet) governance structure. PCORnet, Patient-Centered Clinical Research Network; PI, principal investigator; PI/PD, PI/project director.

The governance structure of the LHSNet is comprised of 3 councils to oversee network processes and functions including the Governance Council, the Workgroup Council, and the Advisory Council (Fig. 2). The Governance Council is the primary governing body of the LHSNet, chaired by the primary principal investigator (PI). Members of the Governance Council include the LHSNet co-PIs, the LHSNet Program Director, site PIs, and patient representatives. The Governance Council meets monthly and reports to PCORI. The Workgroup Council, also chaired by the LHSNet primary PI, oversees the operational activities of the LHSNet with a focus on the completion of project milestones. Members include the LHSNet co-PIs, the LHSNet Program Director, workgroup leaders, site project managers, and site PIs. The Workgroup Council meets biweekly and reports to the LHSNet Governance Council. The Advisory Council informs the LHSNet with membership drawn from organizations external to the LHSNet, including health system leaders, health-care providers, patients, and payers. The Advisory Council enables patients and families, clinicians, researchers, health systems, and payers to ensure that patient needs guide the work, leading to trustworthy and clinically relevant information. The Advisory Council meets annually and informs the direction and priorities of the LHSNet.

Several workgroups have been assembled, including representatives from each participating site, to meet the required deliverables for the network (Fig. 2). The *Regulatory Workgroup* oversees IRB requirements relevant to the multi-organizational research efforts, including streamlining IRB processes, enabling rapid start-up capabilities, and obtaining IRB approvals. The *LHSNet Research Workgroup* leads efforts to define clinical cohorts and integrate research into clinical practice. The *Data Linkage and Data Governance Workgroup* is responsible for enabling EHR and claims data linkages, informing data governance policies, and ensuring data completeness. The *PCORnet (Informatics) Workgroup* is tasked with the major technical aspects of developing the network including development of the following: the PCORnet CDM, the PCORnet Distributed Research Network Architecture, and basic PCORnet query capability. This workgroup is also responsible for data quality, informatics innovation, development of computable phenotypes, and enhancement of the PCORnet CDM. The *Governance and Collaboration Workgroup* leads the development of governing policies for research and collaboration. Finally, the *Engagement Workgroup* is leading efforts to understand existing patient engagement efforts across the network and to establish network-wide patient and stakeholder engagement strategies.

## Milestones

As one of the more recently funded CDRNs in PCORnet, the LHSNet is committed to meet an ambitious set of milestones within the 36-month period of performance. Key milestones, many of which have already been achieved, include the following: streamline IRB requirements across sites through development of a central IRB; increase data linkage for EHR and claims data from 1 million to 3 million lives; demonstrate full functionality of data mapping from each site to the PCORnet CDM; establish PopMedNet Node and demonstrate the ability to execute a basic query across sites; develop computable phenotypes for identifying condition-specific cohorts; validate cases for the 3 established disease cohorts (obesity, heart failure, and osteogenesis imperfecta); and participate in a formally designated PCORnet study.

## Strengths and Limitations

Despite successful completion of milestones in the first 6 months of this project, we anticipate organizational, regulatory, and technical challenges as we ready the LHSNet for patient-centered comparative effectiveness research as part of the national PCORnet infrastructure [5]. The LHSNet, like other CDRNs, is likely to face organizational challenges stemming from the diversity of the participating organizations in terms of organizational and research culture, data management practices, informatics expertise, and experience relevant to participation in large research networks. Frequent meetings of the Governance Council and the Workgroup Council in addition to face-to-face meetings that occur at least biannually ensure that each participating site has ongoing opportunities to bring attention to any challenges or issues. Organizational challenges may also emerge as the LHSNet begins to consider requests for participation in network-level (LHSNet) or national-level (PCORnet) research efforts. The LHSNet has begun to develop formal strategies for prioritizing research within our network, which will move us toward an agreed upon approach and set of standards for considering research opportunities. Standard operating procedures have also been developed for publication of research emerging from LHSNet.

As LHSNet works toward streamlining IRB infrastructure to support multisite research, ethical and regulatory challenges will need to be addressed. Systems will need to comply with the ethical and regulatory requirements of each site and support informed consent and enrollment of patients in an efficient manner across participating sites. Several members of LHSNet bring expertise in clinical research and clinical trials, including centralized IRBs and the identification of eligible patients using EHRs. This expertise will guide our efforts toward a centralized IRB and virtual patient consenting. Currently, members of LHSNet have signed master reliance agreements for Mayo Clinic to serve as the IRB of record. However, by January of 2017, LHSNet will adopt the Streamlined, Multisite, Accelerated Resources for Trials IRB Reliance Platform developed by the National Center for Advancing Translational Science. The National Center for Advancing Translational Science Streamlined, Multisite, Accelerated Resources for Trials IRB reliance model provides flexible resources to LHSNet investigators to harmonize and streamline IRB review through a set of agreements and standard operating procedures that enables each site to cede IRB approval to the institution leading the research effort.

Technical challenges regarding observational research and longitudinal data capture, standardization and harmonization must be addressed by each participating site within LHSNet without disrupting the ways in which clinical data are routinely collected. Each site has successfully populated their DataMart as per the requirements of the CDM and is able to return data in response to data queries. However, individual research projects may require ongoing changes to the CDM. Furthermore, data capture must also be nimble enough to accommodate ongoing changes in clinical practice and related routine data collection. Technical challenges are also likely to be encountered in our planned data linkage efforts, as well as in the development and execution of computable phenotypes. Several members of LHSNet have robust expertise with methods and tools for longitudinal health-care data standardization, data linkage, data privacy, natural language processing, and computable phenotyping from EHRs and administrative claims. This expertise will guide our approach to addressing technical challenges as they emerge.

## Opportunities for Translational Research

The LHSNet is being developed as an LHS, including diverse members across a wide geographic region, to support clinical and translational research. As part of the PCORnet infrastructure, the LHSNet will enjoy the broader reach and potential for impact of the national research network. The PCORnet national data research network is being developed to support participation and data sharing with external investigators who are willing to participate in research studies in conjunction with the PCORI-funded CDRNs. Thus, opportunities for leveraging these networks for clinical and translational science will emerge as the networks mature.

The LHSNet federated approach to an LHS has enormous potential to increase the efficiency with which clinical and biomedical knowledge is created, validated, and translated into practice [7]. As a result, health systems will benefit from the creation of an evidence base for their clinical practice, allowing them to provide the most effective therapies and improve health-care delivery [4]. The LHSNet, through the engagement of patients in the governance of our network and in the research design and implementation, will support research efforts that are meaningful to patients, caregivers, and clinicians. This collaborative effort will ensure that our research is responsive to patients’ needs and enable our health-care systems to achieve clinical outcomes that closely align with patients’ priorities.
